# Management of Hepatocellular Carcinoma in Decompensated Cirrhotic Patients: A Comprehensive Overview

**DOI:** 10.3390/cancers15041310

**Published:** 2023-02-18

**Authors:** Maria Tampaki, George V. Papatheodoridis, Evangelos Cholongitas

**Affiliations:** 1 Academic Department of Gastroenterology, General Hospital of Athens “Laiko”, Medical School, National and Kapodistrian University of Athens, 11527 Athens, Greece; 2 First Department of Internal Medicine, General Hospital of Athens “Laiko”, Medical School, National and Kapodistrian University of Athens, 11527 Athens, Greece

**Keywords:** HCC, decompensated cirrhosis, treatment

## Abstract

**Simple Summary:**

Decompensated patients with hepatocellular carcinoma (HCC) are a wide patient category with limited therapeutic options, and are often excluded from existing trials. Liver transplantation is the best treatment option for such patients but is affected by strict selection criteria and liver donor shortages. The data regarding locoregional treatments in patients with impaired liver function are scarce but indicate a possible survival benefit provided they are well tolerated. Perhaps the systemic treatments, and particularly immunotherapy, are the safest option for such patients based on the available real-life data. Regardless of the type of treatment, close adverse event monitoring is mandatory due to the high risk of hepatic disease deterioration. The aim of this review is to analyze the existing data regarding the administration of treatment in decompensated patients with HCC, evaluate the effect of therapy on overall survival, highlight the potential risks in terms of tolerability and elucidate the optimal therapeutic management.

**Abstract:**

Primary liver cancer is the sixth most common cancer and the fourth leading cause of cancer-related death. Hepatocellular carcinoma (HCC) accounts for 75% of primary liver cancer cases, mostly on the basis of cirrhosis. However, the data and therapeutic options for the treatment of HCC in patients with decompensated cirrhosis are rather limited. This patient category is often considered to be in a terminal stage without the possibility of a specific treatment except liver transplantation, which is restricted by several criteria and liver donor shortages. Systemic treatments may provide a solution for patients with Child Pugh class B or C since they are less invasive. Although most of the existing trials have excluded patients with decompensated cirrhosis, there are increasing data from real-life settings that show acceptable tolerability and satisfying efficacy in terms of response. The data on the administration of locoregional treatments in such patients are also limited, but the overall survival seems to be potentially prolonged when patients are carefully selected, and close adverse event monitoring is applied. The aim of this review is to analyze the existing data regarding the administration of treatments in decompensated patients with HCC, evaluate the effect of therapy on overall survival and highlight the potential risks in terms of tolerability.

## 1. Introduction

Primary liver cancer is the sixth most common cancer and the fourth leading cause of cancer-related mortality worldwide [1,2,3]. Hepatocellular carcinoma (HCC) is the predominant type of primary liver cancer, accounting for 70–85% of all cases [4] and associated with severe morbidity and mortality. Although there are increasing therapeutic options for HCC in patients with compensated liver disease, the management of HCC in patients with decompensated cirrhosis remains challenging and relatively unclear. The International Scientific Guidelines for the management of HCC have analyzed extensively the available therapeutic techniques for patients with Child Pugh (CP) class A or B according to HCC stage, while patients with CP class C are categorized as end-stage liver disease and are eligible only for palliative care or liver transplantation (LT) if they fulfill specific clinical and tumor criteria [5,6,7].

Patients with CP class B are a wide heterogenous patient group with borderline liver function and high-risk for post-treatment hepatic deterioration [8,9]. In particular, they are rarely able to undergo hepatic resection and cannot always receive locoregional treatments due to the risk of liver-related complications [6,10]. Additionally, despite the recent impressive progress in the availability of systemic treatments with different mechanisms of action against HCC, almost all registrational studies have excluded patients with decompensated cirrhosis [11,12,13]. In general, survival rates are considered to be lower in this patient group even after administration of systemic therapies, while the rates of adverse events (AE) are expected to be higher. Regarding HCC patients with CP class C cirrhosis, the treatment landscape is even more unclear since the overall survival (OS) is quite limited for these patients, mostly due to liver-related complications rather than HCC progression [14]. Nevertheless, the administration of HCC treatment to carefully selected patients with CP class C might prolong OS even for this fragile patient group [15].

The aim of this review is to analyze the existing data regarding the administration of treatment for HCC in decompensated cirrhotic patients, evaluate the effect of therapy on overall survival and highlight the potential risks in terms of tolerability.

## 2. Literature Search

A comprehensive literature search was conducted for relevant literature using the “PubMed” database, in which only studies written in the English language published until December 2022 were included. The following search terms were used: “Hepatocellular carcinoma” or “HCC” AND “decompensated cirrhosis” or “Child Pugh B” or “Child Pugh C” AND “liver transplantation” or “locoregional treatments” or “transarterial chemoembolization” or “TACE” or “radiofrequency ablation” or “RFA” or “liver resection” or “systemic treatments” or “immunotherapy” or every drug included in the two last categories. In addition, the references of the research articles were scrutinized for relevant studies.

## 3. Systemic Treatments

The administration of systemic treatments in cases of decompensated cirrhosis remains quite challenging since the safety of such agents has not been confirmed and the consequences from potentially more frequent hepatic toxicity and liver-associated AEs can be dramatic in such patients [16,17]. Consequently, the therapeutic decision should weigh the survival benefit provided by the available therapeutic agents and the risks of further hepatic impairment when the balance is already very fragile. Most clinical trials for systemic treatments do not include CP class B and C patients in order to avoid compromising the clinical outcomes of the studies, thus resulting in lack of scientific evidence to guide clinical management of HCC in this population [18,19].

### 3.1. Multikinase Inhibitors (MKIs) (Table 1)

#### 3.1.1. Sorafenib

Since sorafenib was the only available systemic treatment for HCC patients for almost a decade, the available data on its use in decompensated cirrhosis is possibly richer compared with other systemic treatments (Table 1) [20]. It is the only MKI with an approved indication for administration in CP B class patients [21]. The SHARP trial, the registrational study for sorafenib, did include 20 patients with advanced HCC of CP class B, which was associated with worse overall survival (OS) in the multivariate analysis [22]. The GIDEON study showed that median OS in 3213 real-life patients who received sorafenib for advanced HCC was 13.6 vs. 5.2 vs. 2.6 months in CP class A, B and C patients, respectively [23]. Interestingly, there was a higher incidence of serious adverse events (SAEs) and treatment discontinuation due to AEs in CP class B compared with CP class A patients. Liver- and HCC-related death rates were similar between CP class A and B groups, but significantly higher in the CP class C group. Woo et al. reported that sorafenib treatment was significantly longer in CP class A patients compared with CP class B (233 ± 240 days vs. 100 ± 136 days, respectively; *p* = 0.006) [24]. Liver failure was the most common cause of treatment discontinuation in CP class B patients, as opposed to HCC progression in CP class A patients. In a recent prospective study by Leal et al., the OS was significantly higher in CP class A patients (12 vs. 6 months), but still the reported OS in CP class B patients was considered satisfying enough for this patient group [25]. Finally, a meta-analysis of 30 studies examining the administration of sorafenib as first-line treatment in 8678 patients with advanced HCC further confirmed that OS was significantly worse in patients with CP class B than in class A patients (4.6 vs. 8.8 months, respectively, *p* = 0.001) [26]. However, in the same meta-analysis clinical response, safety and tolerability did not differ significantly between CP class A and B patients. In fact, treatment-related death rates were similar across CP class groups. Based on this, CP class B patients with advanced HCC may respond to sorafenib and tolerate treatment but without prolongation of survival, as they often experience progression of liver disease that leads to treatment discontinuation and decreased life expectancy.

#### 3.1.2. Lenvatinib

Lenvatinib is an MKI approved as a first-line treatment based on the REFLECT study, which compared its efficacy to sorafenib in advanced HCC patients [27]. In a post hoc analysis of the REFLECT study that included CP class A patients and also patients who progressed to CP B during treatment, among the patients who received lenvatinib, the median OS was 6.8 months (95% CI 2.6–10.3) for CP class B patients and 13.3 months (95% CI 11.6–16.1) for CP class A patients [28]. For the sorafenib group, OS was 4.5 months (95% CI 2.9–6.1) for CP class B patients and 12 months (95% CI 10.2–14.0) for CP class A patients. Similarly, the median progression-free survival (PFS) in the lenvatinib group was 3.7 and 6.5 months in CP class B and A patients, respectively. In the same study, the AE rates (number of AE episodes per patient year) for grade 3 treatment-related AE (TRAE) episodes in the lenvatinib arm were 3.65 and 1.41 for CP class B and A patients, respectively, and more patients with CP class B discontinued therapy because of TRAEs (18.3% vs. 7.5% for CP class A). The AEs that most frequently led to lenvatinib dose reduction or discontinuation in the CP class B subgroup were hepatic encephalopathy (15%), decreased appetite (13%), and increased bilirubin (12%). In a retrospective multi-center study by Ogushi et al., the multivariate analysis showed that OS following lenvatinib treatment was significantly associated with CP class (A vs. B, *p* = 0.007) and Barcelona clinic liver cancer (BCLC) stage (BCLC B vs. C, *p* = 0.002) [29]. In particular, OS following 12 months of lenvatinib treatment was 66% and 30% in CP class A and B patients, respectively (*p* = 0.002), while the objective response (OR) rate was markedly higher in CP A5 patients (44%) compared with CP A6 (25.5%), CP B7 (22.2%), and CP B8 patients (5.3%) (*p* = 0.002). Finally, lenvatinib-associated AEs were also found to be higher in the CP class B group and included decreased appetite (*p* = 0.034), diarrhea (*p* = 0.040), vomiting (*p* = 0.009) and increased serum bilirubin levels (*p* = 0.016). In a recent retrospective study that compared the effectiveness of lenvatinib vs. sorafenib treatment in 94 patients with decompensated cirrhosis (CP class B and C), there was no significant difference in the OS between the treatment groups (4.2 in the lenvatinib group vs. 4.1 months in the sorafenib group) [30]. Furthermore, there was no significant difference concerning the AE rates between the regimens. In the real-life setting, the reports regarding the efficacy and safety of lenvatinib in decompensated patients seem to generally agree. In a US study that included 164 patients (49.4% with CP class B cirrhosis), clinical response rates were similar between CP class A and B patients (*p* = 0.11), although dose reductions were higher in patients with CP class B [31]. In terms of OS, a real-life study from Japan including 276 CP class A and 67 CP class B patients showed that the survival rates were 21 and 9 months, respectively. In CP class B patients, drug discontinuation was observed in 47/67 (70%) because of disease progression (*n* = 9), TRAEs (*n* = 36), and worsening of other comorbidities (*n* = 2) [32]. Finally, in a small study by Cosma et al. that included 12 CP A and 14 CP B patients with HCC, the calculated one-year survival rates were 59% and 27%, respectively, and liver disease deteriorated in two CP A and one CP B patient. The most frequent adverse event was fatigue, irrespective of CP status, and in general, the AE rates did not differ significantly between CP groups [33]. In conclusion, lenvatinib, similar to sorafenib, may be efficacious in CP class B patients in terms of HCC response, but higher rates of treatment dose reduction or discontinuation, TRAEs and liver-related deaths limit its effect on OS. Additional data from prospective studies may be needed to clarify if lenvatinib provides a clear benefit in terms of OS in CP class B patients with advanced HCC. 

**Table 1:** Studies examining the efficacy and safety of Multikinase Inhibitors (MKIs) in patients with decompensated cirrhosis and hepatocellular carcinoma.

	Systemic Treatment	Type of Study	Total Patients, *n*	CP B Patients, *n*	CP C Patients, *n*	Mean OS, Months			ORR, %			DCR, %			AEs, %		
						CP A	CP B	CP C	CP A	CP B	CP C	CP A	CP B	CP C	CP A	CP B	CP C
Lencioni 2014 [20]	Sorafenib	Prospective	3213	361	35	13.6	5.2	2.6	n/a	n/a	n/a	n/a	n/a	n/a	82	89	86
Leal 2018 [22]	Sorafenib	Prospective	130	65	0	12	6	n/a	n/a	n/a	n/a	n/a	n/a	n/a	93.8	76.9	n/a
Huynh 2022 [25]	Lenvatinib vs. Sorafenib	Post hoc analysis	478 476	60 47	0 0	13.3 12	6.8 4.5		42.9 12.9	28.3 8.5	-	n/a	n/a	-	10.4 * 11.6 *	18.4 * 19.9 *	-
Ogushi 2020 [26]	Lenvatinib	Retrospective	181	55	0	1-year 66%	1-year 30%	n/a	36.5	16.3	-	n/a	n/a	-	98.4	94.5	-
Tsuchiya 2021 [29]	Lenvatinib	Retrospective	343	67	0	21	9	-	n/a	n/a	-	n/a	n/a	-	n/a	n/a	-
Cosma 2021 [32]	Lenvatinib	Retrospective	28	14	2	1-year 59.3%	1-year 26.9%	n/a	n/a	n/a	n/a	n/a	n/a	n/a	n/a	n/a	n/a
El-Khoueiry 2022 [31]	Cabozantinib vs. placebo	Retrospective	51 22	51 22	0	-	8.5 3.8	-	-	0 0	-	-	57 23	-	-	100 100	-
Bang 2022 [33]	Cabozantinib	Retrospective	110	22	0	9	3.8	-	4.5	0	-	71.5	45.5	-	76.1	72.7	-
Finkelmeier 2021 [34]	Cabozantinib	Retrospective	88	22	0	9.7	3.4	-	n/a	n/a	-	n/a	n/a	-	43.3	72.7	-

CP, Child Pugh; OS, overall survival; ORR, objective response rate; DCR, disease control rate; AE, adverse event; n/a, not available. * Adjusted by patient-years.

#### 3.1.3. Cabozantinib

Data on cabozantinib in decompensated patients with advanced HCC, another MKI agent that can be administered as a second-line HCC treatment, are lately increasing (Table 1) [34]. In a retrospective analysis from the CELESTIAL study focusing on the patients who progressed to CP class B, there was no difference in terms of treatment safety and tolerability in this group compared with the overall population [35]. Moreover, the OS for CP class B patients in the cabozantinb group was 8.5 vs. 3.8 months in the placebo group (HR 0.32, 95% CI 0.18–0.58), and the median PFS was 3.7 vs. 1.9 months (HR 0.44, 95% CI 0.25–0.76), respectively. In a post hoc analysis based again on the CELESTIAL study, the investigators examined the association of albumin-bilirubin (ALBI) grade with OS [36]. The results of this analysis showed that cabozantinib efficacy in terms of OS and PFS was similar between the ALBI grade 1 (score ≤ −2.60) and ALBI grade 2 (score > −2.60 to ≤−1.39) subgroups. Grade 3 AEs associated with hepatic decompensation were found to be higher in the ALBI grade 2 subgroup. However, the survival outcomes were significantly worse for patients with advanced HCC and CP class B cirrhosis (PFS: 4.3 vs. 2.2 months, *p* < 0.001; OS: 9.0 vs. 3.8 months, *p* < 0.001) according to a retrospective Korean study including 110 patients [37]. In the same study, AE rates were generally similar between CP class A and B patients. On the other hand, a recent international real-life study that included 60, 22 and 1 CP class A, B and C patients, respectively, showed that the AE rates were 73% in CP B compared with 43% in CP A patients (*p* = 0.017), and the OS was 7 months in CP B7 vs. 9.7 months in the CP class A group [38]. In contrast, the median OS in CP B8, B9 and C was limited to 3.4 months. The increased AE rate in the CP class B group was attributed to the possible decreased drug metabolism due to impaired liver function, leadingto higher drug levels and thus higher adverse reactions. Based on the above, the effect of cabozantinib on survival of patients with advanced HCC seems to depend significantly on the severity of hepatic impairment, with more favorable outcomes in lower score-CP class B patients. AE and toxicity rates could be limited by administrating lower drug doses provided that efficacy is not compromised.

### 3.2. Immunotherapy (Table 2)

In recent years, immune checkpoint inhibitors (ICIs) have drastically changed the treatment landscape in HCC [39]. CheckMate-040 was the first ICI study that included CP class B patients with advanced HCC [40]. In this phase 1/2 trial, 49 CP class B patients received nivolumab intravenously, resulting in a reported objective response rate (ORR) of 12% and a disease control rate (DCR) of 55%. Interestingly, the safety profile of nivolumab in this population was comparable to the CP class A patients, as TRAEs were reported in 51% of patients and led to discontinuation in 4% (83% and 6% in the CP class A patients, respectively) [41]. These results were further confirmed by a recent real-life study that recruited 431 CP class B patients from the Veteran Affairs medical centers in the USA to compare efficacy and safety of sorafenib vs. nivolumab [42]. The median OS was 5 months for the 79 patients that received nivolumab vs. 4 months for the sorafenib group, and treatment was discontinued due to toxicity in 12% of patients receiving nivolumab compared with 36% receiving sorafenib (*p* = 0.001). A Korean study by Choi et al. showed that ORR with nivolumab treatment was significantly lower in CP class B compared to CP A patients (2.8% vs. 15.9%; *p* = 0.010) and that OS was specifically worse in patients with CP B8 or B9 than in those with CP B7 (7.4 vs. 15.3 weeks; *p* < 0.020) [43]. Additionally, AE rates were similar between the CP class A and B groups, and immune-mediated AEs were more frequent in CP class A patients (2 patients with hepatitis and 3 with pneumonitis vs. 0 patients in the CP class B group). Lower response rates in the CP class B group in this study were explained by the reduced capacity of immune response because of advanced cirrhosis. The same explanation was provided for lower immune-mediated adverse events in the CP class B population. Additionally, a real-life study examining the efficacy and safety of nivolumab or pembrolizumab treatment in 32 CP class A and 28 CP class B patients with advanced HCC showed that the ORR and DCR for CP class A vs. B was 9% vs. 14% (*p* = 0.438) and 56% vs. 46% (*p* = 0.947), respectively [44]. Median OS values of 16.7 (95% CI, 8.2–25.2) months for CP class A and 8.6 (95% CI, 4.8–12.4) months for CP class B (*p* = 0.065) were reported, whereas there was no significant difference in terms of AE rates (CP class A vs. B, 31% vs. 43%; *p* = 0.352) or high-grade AEs (CP A vs. B, 16% vs. 18%; *p* = 1.000).

Concerning first-line treatments, atezolizumab-bevacizumab is a combination with high efficacy in non-resectable HCC based on the IMbrave 150 study results [5,45]. A recent multi-center retrospective study by D’Alessio et al. examined the safety and efficacy of the combination treatment in 154 CP class A and 48 CP class B patients [46]. According to this study, the treatment-related AE rates were similar between the two groups, while ORR and DCR were 25% and 73%, respectively, without significant difference across CP classes (Table 1). However, the median OS was 16.8 months (95% CI, 14.1–23.9) for CP class A patients, vs. 6.7 months (95% CI, 4.3–15.6) for patients with CP class B (*p* = 0.0003). This difference was attributed to deaths due to the underlying liver impairment. Interestingly, TRAEs and bleeding events were also comparable between the CP groups. Interestingly, Chen et al. examined the OS of patients after failure of first line treatment with atezolizumb/bevacizumab. All the patients had CP A cirrhosis at treatment initiation, but 15% and 17% of them progressed to CP B and C, respectively, after failure. The median OS after treatment discontinuation was 9.6 vs. 3.8 vs. 1.2 months, for CP A, B, and C patients, respectively. After therapy failure, the tumor burden was increased, and 32% of the patients did not maintain CP A liver reserve. Not receiving second-line treatment was associated with liver deterioration and poorer OS [47]. Moreover, Persano et al. compared efficacy of atezolizumab/bevacizumab vs. lenvatinib in HCC patients with HCC and showed that ORR was similar for CP B patients (22% vs. 36%, respectively, *p* = 0.43). In the same study, the ORR rates for CP A patients were 28% for the atezolizumab/bevacizumab group and 39% for the lenvatinib group [48]. Another real-life study by de Castro et al. reported that the median OS was 12 months (95% CI: 8.2–15.8), 6.8 months (95% CI: 3.1–10.5; *p* = 0.04) and 1 month (95% CI: 0.0–3.9; *p* < 0.001) for CP class A, B and C patients, respectively [49]. Apart from the CP score, the ALBI score was also significantly associated with OS. Moreover, patients with ALBI grade ≥2 (*p* = 0.002) and decreased performance status (*p* < 0.001) at baseline were at highest risk for developing ascites and hepatic encephalopathy. Intriguingly, mono-immunotherapy was not associated with the above adverse events. Based on the previous findings, immunotherapy may be a relatively safe option for patients with impaired liver function, although close monitoring is mandatory, especially when it is combined with anti-VEGF agents. The combination of two ICI agents, durvalumab plus tremelimumab, has also been tested in patients with unresectable HCC and has yielded positive results in terms of OS, but the majority of the study population were CP A patients [50]. The fact that ICIs metabolism does not depend on liver function possibly provides an advantage for CP class B patients. Furthermore, ICIs especially when combined with anti-VEGF agents, seem to offer a survival benefit that may not be as high as that offered in CP class A, but still, they may prolong life expectancy in carefully selected CP class B patients (Table 2). In other words, the unmet need that exists for CP class B patients with advanced HCC could be covered by this type of treatment in the future.

**Table 2:** Studies examining the efficacy and safety of Immune Checkpoint Inhibitors (ICIs) in patients with decompensated cirrhosis and hepatocellular carcinoma.

	Systemic Treatment	Type of Study	Total Patients, *n*	CP B Patients, *n*	CP C Patients, *n*	Mean OS, Months			ORR, %			DCR, %			AEs, %		
						CP A	CP B	CP C	CP A	CP B	CP C	CP A	CP B	CP C	CP A	CP B	CP C
Kudo 2021 [36]	Nivolumab	I/II phase	49	49	0	-	7.6	-	-	12	-	-	55	-	-	51	-
Chapin 2022 [38]	Sorafenib vs. Nivolumab	Retrospective	439 79	439 79	0	-	4 5	-	-	n/a	-	-	n/a	-	-	n/a	-
Choi 2020 [39]	Nivolumab	Retrospective	203	71	0	10	3.6	-	15.9	2.8	-	42.4	22.5	-	n/a	n/a	-
Scheiner 2019 [40]	Nivolumab vs. Pembrolizumab	Retrospective	65	28	5	16.7	8.6	n/a	9	14	n/a	56	46	n/a	31	43	n/a
D’Alessio 2022 [42]	Atezolizumab/bevacizumab	Retrospective	216	48	0	16.8	6.7	-	26	21	-	74	68	-	48	46	-
de Castro 2022 [43]	Atezolizumab/bevacizumab	Retrospective	147	35	6	12	6.8	1	n/a	n/a	n/a	n/a	n/a	n/a	n/a	n/a	n/a
Persano 2022 [47]	Atezolizumab/bevacizumab vs. lenvatinib	Retrospective	823 1312	n/a	n/a	n/a	n/a	n/a	27.7 38.8	22.2 36.3	-	n/a	n/a	n/a	n/a	n/a	n/a

CP, Child Pugh; OS, overall survival; ORR, objective response rate; DCR, disease control rate; AE, adverse event; n/a, not available.

## 4. Locoregional Treatments (Table 3)

There is no doubt that LT is the optimal treatment option for decompensated patients with HCC tumors within the Milan criteria [5,6]. However, the low availability of liver transplants in many cases requires alternative therapeutic strategies for patients with advanced liver disease. Based on the current International Guidelines for the management of HCC, patients with CP class B can benefit from local ablation treatments (LAT), trans-arterial chemoembolization (TACE) or transarterial radioembolization (TARE) based on their BCLC status, provided that their liver function is adequately preserved and the risk for hepatic disease deterioration is not high [5,6]. Especially for patients with CP B8 or B9, when surgical treatments are not applicable due to the risk of hepatic deterioration, the less invasive locoregional techniques may provide survival benefit [51]. However, the availability of studies for the administration of locoregional treatments in patients with CP class C cirrhosis and their effect on OS is restricted to some small retrospective trials (Table 3).

### 4.1. Local Ablation Treatments (LAT) 

For patients with CP class A or B and small single tumors < 3 cm who are not eligible for surgery, radiofrequency ablation (RFA) is the indicated treatment option [6]. However, according to a meta-analysis by Casadei-Gardini et al., CP class B is predictive of poor OS and RFS (*p* < 0.0001) compared with CP class A in HCC patients treated with RFA alone [52]. Regarding the effect of RFA on decompensated patients, a retrospective study in 19 patients with mean CP score 10.7 (range 10–12) showed a median survival time of 12.0 ± 1.7 months and reported two deaths of hepatic failure, one at two months and one at four months after treatment [53]. Another study that examined the effects of non-surgical treatments in HCC patients with CP class C reported zero survival benefit of LAT, possibly because hepatic deterioration progressed faster than HCC [54]. On the other hand, Kudo et al. showed that the administration of non-transplant techniques in HCC patients with CP class C who exceeded the Milan criteria or could not be transplanted due to liver donor shortage was a significant prognostic factor of better survival compared to non-treated patients [55].

### 4.2. TACE

TACE is currently the indicated treatment for patients with intermediate stage HCC (BCLC B) and CP class A or B [5,6]. Since it is a therapeutic technique based on the trans-arterial administration of chemotherapeutic agents and the occlusion of the tumor-feeding vessel, it has been associated with deterioration of the hepatic function and decompensation when applied to patients with advanced liver disease [56,57]. Therefore, several prognostic models have been developed in order to select carefully the patients that are suitable to receive TACE without risking their hepatic reserve [58,59]. These models are based on biochemical parameters, ALBI score, CP score and tumor characteristics, among others. In a large study by Takayasu et al. that included 4966 HCC patients who underwent TACE, the survival rate was significantly associated with CP score, with the lowest rates being reported in CP class B patients with three lesions ≥ 5.1 cm [60]. Specifically, the 3-year survival rate was 53% in CP class B patients with a single tumor (vs. 73% in CP class A, *p* = 0.0001) and 22% in patients with ≥4 tumors (vs. 46% in CP class A, *p* = 0.0001). Treatment-related death occurred in 19 (0.38%) out of 4966 patients (10/3229 CP class A, 8/1296 CP class B and 1/167 CP class C). In another study including 100 CP class A and 90 CP class B/C cirrhotic patients, the administration of TACE was equally efficacious in both groups in terms of tumor necrosis, but the OS was significantly higher in the CP class A group (21.9 vs. 13.7 months, *p* = 0.03) [61]. There was no significant difference in terms of 30-day or 90-day post-treatment mortality between the two groups. The survival curves for the two CP groups separated at 3 months and reached maximal separation at 12 months. According to the Cox proportional hazards model, post-treatment mortality for the CP class B group was significantly associated only with total tumor diameter (hazard ratio 1.26, 95% CI 1.10–1.44, *p* < 0.001). According to Piscaglia et al., TACE treatment offered a survival rate of 22 and 8 months in patients with CP B7 and B8, respectively [51]. The study concluded that TACE should be performed in CP class B patients with compensated disease (B7), but it could be detrimental for liver function in patients with more advanced hepatic impairment. According to the above, it can be assumed that even within the group of CP class B patients, the OS rates may differ significantly based on the exact CP score, so TACE is probably a good therapeutic option for those who have borderline liver function and BCLC B HCC. 

The available studies examining the administration of TACE specifically to CP class C patients are very limited due to the mentioned risks. In a retrospective study comparing best supportive care (BSC) to locoregional treatments in CP class C patients with multinodular HCC, the OS rates were found to be significantly higher in patients treated with TACE compared with non-treated patients (14 vs. 2 months, respectively, *p* < 0.0001) [62]. In the same study, the propensity score matching for patients with tumors within the Milan criteria showed no significant differences in the clinical characteristics between the two treatment groups, so it was concluded that the patient selection bias was relatively low. Similarly, in the above-mentioned study of Kudo et al., superselective TACE in decompensated patients with multinodular HCC provided better survival compared with palliative care [55], which is the currently recommended treatment for such patients. However, the most benefited patients in this study were those with the lowest CP C scores, while most patients with 14- or 15-point scores remained untreated. In another Japanese study by Nouso et al., the OS rates after superselective TACE were superior to BSC in matched CP C patients (*p* < 0.009) [54]. Regarding the selection of decompensated patients eligible for TACE in this study, the therapeutic technique was specifically performed in patients without severe portal vein thrombus, with median bilirubin of 1.9 mg/dL and median prothrombin time of 64%. Consequently, TACE could prolong OS in carefully selected patients with CP C scores in specialized centers performing superselective embolization. However, it is crucial to understand the limitations of this therapeutic technique for patients with advanced liver disease and HCC and seek for other therapeutic options, such as systemic treatments, when required. The timely switch to a more tolerable therapeutic option for patients with higher CP scores such as immunotherapy, even in earlier HCC stages, could benefit decompensated patients who are not eligible for more invasive treatments.

### 4.3. TARE

TARE, also called selective internal radiation therapy (SIRT), is another locoregional technique based on the transarterial administration of microspheres loaded with radioactive compounds such as yttrium-90 or lipiodol labeled with iodine^131^ or rhenium^188^ [63]. The indication of this treatment for HCC patients is not clearly defined but it is mostly administered in patients with BCLC stage B or C [64]. One main advantage of TARE in terms of safety is the absence of macro-embolic effects, so it does not affect hepatic blood flow, which is beneficial for patients with advanced liver disease, especially in cases of macrovascular invasion [65]. However, several AEs are reported following TARE, such as liver failure or radio-induced liver disease (4%), biliary complications (<10%) and post-radioembolization syndrome (20–55%). 

**Table 3:** Studies examining the efficacy and safety of locoregional treatments in patients with decompensated cirrhosis and hepatocellular carcinoma.

	Treatment	Type of Study	Total Patients, *n*	CP B Patients, *n*	CP C Patients, *n*	Mean OS, Months			ORR, %			DCR, %			AE %		
						CP A	CP B	CP C	CP A	CP B	CP C	CP A	CP B	CP C	CP A	CP B	CP C
Kim et al. [53]	RFA	Retrospective	19	0	19	-	-	12	-	-	88.5%	-	-	n/a	-	-	n/a
Nouso et al. [54]	LAT	Retrospective	23	0	23	-	-	1-year 69.1%	-	-	n/a	-	-	n/a	-	-	n/a
Kudo et al. [55]	RFA	Retrospective	60	0	60	-	-	1-year 67%	-	-	n/a	-	-	n/a	-	-	n/a
Takayasu et al. [60]	TACE	Prospective	4966	1296	167	1-year 61%	1-year 43%	1-year 23%	n/a	n/a	n/a	n/a	n/a	n/a	n/a	n/a	n/a
Dorn et al. [61]	TACE	Retrospective	190	90		21.9	13.7		79%	84%		n/a	n/a		n/a	n/a	n/a
Piscaglia et al. [51]	TACE	Retrospective	86	86	0	-	21	-	-	n/a	-	-	n/a	-	-	n/a	-
Nouso et al. [54]	TACE	Retrospective	27	0	27	-	-	1-year 62.5%	-	-	n/a	-	-	n/a	-	-	n/a
Kudo et al. [55]	TACE	Retrospective	79	0	79	-	-	1-year 69%	-	-	n/a	-	-	n/a	-	-	n/a
Zu et al. [66]	TARE	Retrospective	106	27	0	20.2	5.5–6	-	-	-	n/a	-	-	n/a	-	-	n/a
Abouchaleh et al. [67]	TARE	Retrospective	185	60	32	13.3	6.9	3.9	n/a	n/a	n/a	n/a	n/a	n/a	8%	8%	32%
Memon et al. [68]	TARE	Retrospective	63	35	0	13.8	6.5	-	37%	32%	-	37%	57%	-	n/a	n/a	-

RFA, radiofrequency ablation; TACE, transarterial chemoembolization; TARE, transarterial radioembolization; CP, Child Pugh; OS, overall survival; ORR, objective response rate; DCR, disease control rate; AE, adverse event; n/a, not available.

In a study by Zu et al. [66] that examined the effect of TARE across CP groups and included a total of 106 patients with BCLC stage C, it was shown that the median OS was 20.2 months for patients with CP A class, 6 months for CP B7 and 5.5 for CP B8/9 groups. Despite the assumed lower risk of liver ischemia with TARE, this did not translate into clinical survival benefits for CP B7 in the study. On the contrary, since the majority of patients had impaired liver function due to portal vein invasion, the authors concluded that the severity of the hepatic disease had a greater impact on OS than the tumor burden. In another study that included 185 HCC patients with portal vein thrombosis, the median OS was 13.3 months for CP A class patients, 6.9 months for CP B7 and 3.3 for CP ≥ 8 groups [67]. Interestingly, when the latter group of patients was stratified by location of portal vein thrombosis, the median OS was 8.4 months for segmental, 4.4 months for lobar, and 3.4 months for main portal vein thrombosis (*p* = 0.015). In the multivariate analysis, ECOG status and extent of portal vein thrombosis were significant predictors of survival, but CP status was not. Additionally, for patients with CP B7 and CP B8 class, tumor size (>5 cm) was associated with worse survival. Finally, in a study by Memon et al., survival following TARE was 13.8 months for the group with CP A and 6.5 months for CP B7 class patients [68]. At the time of HCC progression, 50% of patients who had CP B7 scores at baseline had progressed to CP score ≥ 8, while a similar percentage (55%) of CP A score patients had progressed to CP B. In conclusion, TARE could be a safer choice for HCC patients with borderline hepatic function (CP B7 scores), especially with macrovascular involvement, although its benefit in patients with more advanced cirrhosis is still debatable. Furthermore, the extent of portal vein thrombosis seems to affect significantly patients’ survival [67].

## 5. Surgical Treatments and Liver Transplantation

HCC surgical resection in eligible patients with very early or early tumor stage may lead to a 5-year survival rate of 50–68% in specialized centers [69,70,71]. However, impaired liver function and severe portal hypertension are the most common contraindications for hepatic surgery in cirrhotic patients, since they are associated with significant post-operative morbidity and liver decompensation [72,73]. The selection of patients that can tolerate surgery with minimal risk for hepatic deterioration is based on CP/MELD scores and the evaluation of the functional capacity of the future liver remnant [72,74]. Liver function tests, such as the LiMax test, which is based on the hepatocyte-specific metabolism of the ^13^C-labelled substrate by the cytochrome P450 1A2 enzyme, have been developed as surrogate parameters of liver function capacity to guide treatment decisions [75]. In recent years, patients with portal hypertension and low risk for hepatic decompensation have been carefully selected based on an algorithm that predicts the exact risk for liver deterioration [76]. Laparoscopic techniques have further extended the criteria for choosing patients who are able to withstand hepatic resection without severe morbidity [77,78]. In fact, a recent study by Azoulay et al. showed that cirrhotic patients with hepatic venous pressure gradient ≥ 10 mmHg can undergo laparoscopic liver resection with low rates of mortality and hepatic decompensation [75]. However, almost all patients of the study were compensated with CP class A and a median MELD score of 8. In other words, the criteria for the eligibility of HCC patients for hepatic surgery may have been widened, but still the possibility to include patients with CP class B/C is out of the question.

Liver surgery may be the optimal choice for compensated patients with HCC [79], but LT remains the best option for those with CP class B or C provided that they fulfill the strict transplantation criteria [80,81]. As a therapeutic technique, LT not only removes the tumor, but also offers a cure for the underlying hepatic disease. The main limitation of this curative treatment is the shortage of liver donors and consequently the frequent dropout of HCC patients from the transplantation lists due to disease progression [82]. Rigorous patient selection based on specific clinical and tumor criteria (MELD score and Milan/San Francisco criteria) and waiting time in transplant list < 6 months are pivotal to achieve minimal HCC recurrence rates and maximize OS [79]. Locoregional therapeutic techniques have been administered as a bridge to transplantation in order to downstage the tumor or delay HCC progression [83,84]. However, patients with decompensated cirrhosis may not be eligible for these treatments due to the risk of hepatic disease deterioration, and thus, they should be prioritized based on their MELD score [80]. Recently, there have been increasing reports on the use of systemic treatments such as sorafenib or cabozantinib as bridging therapies for HCC patients before LT [85,86]. ICIs have also been used as neo-adjuvant treatment before LT, but perhaps their administration should be paused at least 3 months before transplantation in order to avoid the risk of graft rejection after LT [80]. If transplanted within the Milan criteria, patients with HCC have excellent OS rates with an estimated 5-year survival close to 60–75% [87,88,89]. In conclusion, LT represents the best treatment choice for HCC patients with CP class B or C scores and tumor characteristics within the Milan criteria (or the extended UCSF criteria), but the low availability of liver grafts and the high dropout rates or deaths within the transplant lists indicate the necessity of alternative/bridging treatments to optimize OS rates for such patients.

## 6. Palliative Care

Unfortunately, 15–20% of HCC patients present with end stage HCC and an estimated median survival close to 3–4 months [90]. This patient category consists not only of patients with a high tumor burden and metastatic disease but also of patients with CP class C and affected physical performance status. The available therapeutic techniques cannot offer survival benefit for this patient group, so the healthcare services that can be provided focus on management of the complications of cirrhosis and pain, nutrition and psychological support [91].

## 7. Discussion

Patients with CP class B or C cirrhosis are a heterogenous group with various clinical characteristics, different degrees of performance status and hepatic function [92]. Therefore, the decision for HCC treatment should be based on individual patient status according to liver function scores such as CP, MELD and ALBI scores, and of course, the tumor stage of each patient [50]. Based on our research, the existing clinical data on the safety and efficacy of HCC treatments for this patient group are scarce, but they generally show that OS is lower compared with CP class A patients, regardless of treatment, while TRAEs are more frequent and can lead to hepatic deterioration (Table 4). Immunotherapy seems to be the safest option among systemic treatments since it has not been associated with liver-related AEs in the existing studies [42,43,45]. Recent data examining the sequential use of ICIs (first-line and second-line) in compensated patients with HCC have shown promising results in terms of efficacy without high-grade TRAEs [93]. Since immunotherapy is the safest option among systemic treatments for decompensated patients, ICI rechallenge in this patient group should be studied as it may offer additional therapeutic choices.

For HCC patients with small HCC (BCLC 0/A) and CP class B or C cirrhosis, the therapeutic management clearly leans towards LT, as it provides the highest OS rates [87,88]. With regards to other surgical techniques, a few patients with CP class B cirrhosis and well-compensated disease may benefit from laparoscopic surgery if chosen properly [70,76,77]. Since RFA is a minimally invasive technique with low risk for liver related AEs, it can also prolong OS for the patients with early HCC who cannot undergo surgery and can also be a bridging therapy until a liver transplant is available [52]. Moreover, it can be an alternative option for patients with small tumors that are not eligible for LT [54]. However, its effect on the OS of patients with CP class C is under debate, and consequently, patients of this category should be selected very carefully in specialized centers. 

Intermediate HCC (BCLC B) is the classic indication for TACE, especially in patients with adequate hepatic function [6]. As mentioned above, a few studies have shown that TACE may prolong OS, even in decompensated patients, when compared with palliative care [50,54]. Perhaps carefully selected patients with lower scores within CP class B groups and clinical characteristics that permit the administration of TACE may be benefited in specialized centers. 

TARE is also an available option perhaps suitable for patients who cannot undergo TACE due to portal vein invasion [67] or as an intermediate treatment between locoregional therapies and systemic agents [68]. Its non-occlusive effect may be an asset for patients with borderline liver function [65], while its role as a bridge therapy to LT is also under discussion.

For those who do not respond or cannot undergo TACE, systemic treatments are the next available therapeutic option. According to small, mainly retrospective studies, systemic therapies may provide a survival benefit for patients with intermediate or advanced HCC and decompensated cirrhosis with relatively adequate tolerability [24,30,34]. Of course, larger prospective studies are needed to further support these indications.

In general, OS rates seem to be significantly lower in decompensated patients with HCC compared with Child Pugh A class patients, mainly due to hepatic disease progression and liver-related mortality. Therefore, the benefit of the administrated HCC treatment is not always clear since the prolongation of survival is relatively low. Perhaps ORR and PFS rates are a more accurate reflection of the efficacy of the provided therapies and their effect on HCC patients as they focus on the immediate impact of anti-cancer treatment on the disease course. 

## 8. Conclusions

Although the International Guidelines and the registrational studies of HCC treatments focus on patients with compensated cirrhosis, decompensated patients are a large population with heterogenous clinical characteristics and limited therapeutic options. HCC treatment should be individualized for this fragile patient group in order to provide maximum survival with minimal risk for liver toxicity. Good performance status should be recognized as a possible indication for treatment administration despite liver impairment. The use of specific scores for the evaluation of hepatic function and the assessment of TRAE risk may also support clinical decision making. Additionally, the identification of the correct parameters of treatment response such as ORR or PFS instead of OS may improve the therapeutic management. Nevertheless, the inclusion of patients with CP class B or C in larger studies is needed to clarify to what extent these patients can benefit from the available anti-cancer treatments. It is possible that the treatment landscape will evolve drastically in the coming years since the number of the latest clinical trials that include this patient category is significantly increasing.

## Figures and Tables

**Table 4:** Advantages and disadvantages per treatment category in patients with decompensated cirrhosis and hepatocellular carcinoma.

Type of Treatment	Advantages	Disadvantages
Systemic therapy	Acceptable tolerance and safety Bridging therapy to liver transplantation * Alternative therapeutic option when locoregional treatments are not tolerated	Lower OS rates in CP B compared with CP A patients More frequent AEs in CP B compared with CP A Scarce data in CP C patients
Radiofrequency ablation	Minimally invasive Low AE rates Bridging therapy to liver transplantation	No clear benefit for OS in CP C patients
Transarterial Chemoembolization	Bridging therapy to liver transplantation May prolong OS compared with palliative care in decompensated patients	Risk of hepatic deterioration Specialized center required CP C patients are rarely eligible
Transarterial radioembolization	Safer in cases of portal vein thrombosis	Risk of hepatic deterioration CP C patients not eligible
Liver resection	High OS rates Laparoscopic techniques are more tolerated Can be applied in selected patients with portal hypertension	Restricted to CP A patients
Liver transplantation	Best available therapeutic option Optimal OS	Strict criteria Liver donor shortage Frequent drop-out from the transplantation list due to HCC progression

OS, overall survival; CP, Child Pugh; AE, adverse event; HCC, hepatocellular carcinoma. * Caution that immunotherapy may be associated with higher rejection rates after liver transplantation.
